# Advances and future directions in identifying specific taxa from microbial meta-omics data: from pipeline to deep learning

**DOI:** 10.1128/msystems.00800-25

**Published:** 2026-04-30

**Authors:** Jingkang Zhang, Xingjie Wang, Di Wang, Zikui Zheng, Hongmei Wang, Liyuan Ma

**Affiliations:** 1 Institute of Natural Resources Survey, China University of Geosciences 12564, Wuhan, China; 2 MOE Key Laboratory of Groundwater Quality and Health, School of Environmental Studies, China University of Geosciences 504988 , Wuhan, China; 3 Key Laboratory of Mine Ecological Effects and Systematic Restoration, Ministry of Natural Resources, Beijing, China; University of California, San Diego, La Jolla, California, USA

**Keywords:** specific taxa identification, meta-omics, deep learning, high-throughput sequencing, explainable AI

## Abstract

Molecular profiling enabled by meta-omics technologies has significantly expanded our knowledge of microbial catalog across diverse environments. Increasing attention has now been focused on identifying ecologically significant taxa, particularly keystone that stabilize communities, rare taxa that underpin functional redundancy, and indicators that reflect environmental gradients. However, current pipeline methods remain limited in deciphering complex ecological relationships and modeling the evolution of community dynamics. As a transformative computational tool, deep learning (DL) offers novel strategies to address these challenges through autonomous feature extraction, nonlinear interaction modeling, and integration of multi-modal data sets. Nevertheless, there are still obstacles to the widespread adoption of DL for collaborative identification of specific microbial taxa, primarily including the intrinsic heterogeneity and imbalance of data sets, the difficulty of model generalization across diverse ecosystems, and the limited ecological interpretability of model outputs. This review summarizes existing research advances and proposes to build a unified DL framework for multi-modal data, exploring its implementation pathways, challenges, and potential coping strategies. The envisioned framework establishes a multi-task learning architecture for unified identification of keystone, rare, and indicator taxa, incorporating domain knowledge through ecological constraint layers and explainable AI modules, while providing flexible implementation pathways for heterogeneous data integration and model customization across microbial ecosystems. This framework has the potential to form a closed-loop verification in combination with synthetic microbial community experiments, reshape the paradigm of microbial community research, and promote the transition from empirical classification to mechanistic ecological cognition.

## INTRODUCTION

Microbial communities play crucial roles in global biogeochemical cycles and host–microbe symbiosis, being widely distributed across diverse environments ranging from the cryosphere to the deep lithosphere (1, 2). Although culturomics has made significant strides in specific ecosystems in recent years (for instance, over 50%-70% of microbes in the human gut are now culturable), the vast majority of microbes in broader complex environments remain unculturable (e.g., typically less than 1%–5% in soil and marine environments), persisting in an “invisible” state (3–5). These uncultivable microbes, along with silent functional genes and uncharacterized taxa, are collectively referred to as “microbial dark matter” (6, 7). Despite their undisputed ecological significance, the investigation of this vast, untapped repertoire of microbial diversity and metabolic potential has been hampered by methodological limitations (8–10).

Advances in high-throughput sequencing technologies, such as 16S rRNA gene amplicon sequencing, genome-resolved metagenomics, and functional genomics, have markedly enhanced our capacity to investigate microbial diversity and community composition in a variety of ecosystems (11–13). As microbial ecology continues to evolve toward a function-oriented and ecosystem holistic perspective, taxa that exert disproportionate ecological effects within communities have attracted growing attention (2, 14). In particular, keystone taxa that maintain community structure and stability (15), rare taxa that underpin functional redundancy (16), and indicator taxa that reflect environmental gradients (17). Crucially, these functional categories are not mutually exclusive in biological systems; their intersections often harbor taxa of unique functional significance (18). For instance, rare keystone, despite their scarcity, exert disproportionate influence on community stability, acting as “hidden drivers” of the ecosystem (16, 18). Furthermore, taxa sharing all three attributes simultaneously maintain network topology and reflect environmental shifts, serving as “critical ecosystem barometers” (19–21). Therefore, elucidating the ecological roles, interrelationships, and impacts of these specific taxa on community dynamics is becoming an important direction in microbial ecology research (22).

Although the emergence of high-throughput sequencing technology and the launch of large-scale studies such as the International Microbiome Projects have accelerated the rapid accumulation of massive microbiome data sets (23–25), the exponential growth of microbiome sequencing data has also introduced significant computational and analytical challenges (26). Traditional methods that rely on linear assumptions and manual feature selection struggle to accommodate the high dimensionality, sparsity, and zero-inflation characteristic of microbiome data sets (27, 28). Moreover, the highly nonlinear, hierarchical structures and complex interactions within microbial communities further limit the applicability of these conventional approaches in capturing community assembly patterns across habitats and disentangling ecological dependencies (29, 30). Data-driven methods such as machine learning (ML) and deep learning (DL) offer the ability to automatically extract features and model nonlinear relationships (22, 31). Especially, DL excels at learning hierarchical representations and detecting subtle ecological interaction patterns, making it a powerful analytical tool in microbial ecology (32, 33).

Based on the development of high-throughput sequencing technology and the attendant challenges in data analysis, this article discusses the current status of research on three types of taxa with specific ecological significance and key issues that remain unresolved and summarizes the applications and limitations of classical ML methods in microbial ecology research. Furthermore, we analyze the current applications and challenges of DL in identifying microbial taxa and highlight the transformative role of DL in advancing this field. The aim of this review is to provide insights into the construction of a unified DL framework that enables more accurate and comprehensive microbial taxa identification, facilitating a deeper understanding of microbial community structure and ecosystem functions.

## IDENTIFYING SPECIFIC MICROBIAL TAXA FROM META-DATA: CURRENT STATUS AND CHALLENGES

High-throughput sequencing technologies have revolutionized microbial ecology by generating comprehensive and cultivation-independent data sets that capture both taxonomic composition and functional potential across diverse habitats (26, 29, 34). 16S rRNA gene amplicon sequencing rapidly obtains taxonomic information of microbial communities from diverse ecosystems (e.g., soils, marine environments, and the human gut) by targeting conserved regions interspersed with hypervariable segments, revealing numerous previously undetectable microbial taxa and substantially expanding our understanding of microbial diversity and ecosystem dynamics (24, 25, 35). In addition, functional genomics connects cultured isolates to their genetic and functional properties (36), and metagenomics reconstructs complete gene catalogs and metabolic pathways directly from environmental samples (24, 37). Collectively, these methods provide a rich meta-omics resource that allows researchers to move beyond simple taxa inventories toward identifying ecologically important microbial taxa.

In highly diverse microbial communities, ecologists classify certain taxa as ecologically significant due to their disproportionately large roles. Identifying specific microbial taxa that play critical ecological roles, particularly keystone, rare, and indicator taxa, is essential for a deep and comprehensive understanding of microbial community ecology. Keystone is classically defined as taxa that exert a disproportionately large influence on their environment relative to their abundance (19). Since Paine first introduced the concept of keystone taxa in 1969 (38), its precise ecological definition has been a longstanding subject of debate and has continually evolved over the years (39). Co-occurrence network analysis, a commonly employed method for identifying keystone taxa, investigates potential microbial interactions by examining abundance correlations within sequencing data, thereby constructing microbial association networks (30, 40). In these networks, putative keystone taxa often emerged as highly interconnected hub nodes or exhibited high centrality measures, suggesting extensive interactions with other community members (41). Such network-based scoring has been widely used to nominate candidate keystone taxa across various ecosystems, ranging from soil to human gut habitats (15, 22, 42). However, network correlations only provide suggestive insights and do not necessarily imply causation, while spurious associations (e.g., co-occurrence due to shared habitat preferences rather than direct interactions) may mislead interpretations (43). Moreover, co-occurrence networks are highly sensitive to the inherent noise and sparsity in microbial data sets (44), leading to false-positive correlations and overlooked interactions (45), particularly involving rare taxa (46).

High-throughput sequencing reveals that, in addition to a few dominant taxa, most communities contain organisms detected at very low frequencies (16). These rare taxa can number in the hundreds or thousands within a single sample, representing a vast reservoir of biodiversity collectively known as the rare biosphere (47, 48). Traditional studies have often overlooked the roles of rare taxa; however, in recent years, rare taxa are increasingly recognized as critical yet vulnerable components of ecosystems (49–51). Despite their low abundance, rare taxa significantly contribute to functional redundancy and serve as a potential reservoir for ecological resilience (16, 18). They persist in small numbers until environmental conditions become favorable for their growth, at which point some rare taxa rapidly proliferate, performing crucial biogeochemical transformations when needed, such as in response to specific substrates or environmental conditions (e.g., pollutants or changes in pH) (52, 53). Nevertheless, many researchers use arbitrary thresholds (e.g., taxa with relative abundances below 0.1% or 0.01% within samples) to define the rare biosphere (54, 55). These thresholds vary considerably and may lack universality across different habitats and ecosystems (56, 57). Such empirical categorization involves subjective assumptions and risks neglecting the dynamic characteristics of rare taxa in specific communities (58, 59). The inability to capture this dynamic nature has constrained advancements in identifying and studying rare taxa within the broader ecological context (60). Additionally, sequencing depth is equally critical, as low-abundance taxa may be missed or discarded as noise in shallow sequencing efforts (26, 61, 62).

Although indicator taxa may not drive community processes, their abundance patterns, such as presence, absence, or fluctuations, sensitively reflect changes in specific environmental conditions (14, 24, 63), offering valuable insights into habitat states (64). Due to their rapid responses to perturbations and relative ease of sampling (e.g., via water or soil samples or non-invasive body swabs), microbes represent attractive early-warning indicators (14, 65, 66). To identify indicator taxa, researchers typically adapt classical community ecology techniques to high-throughput sequencing data (67, 68). One prominent method is Indicator Value (IndVal) (69), which quantitatively evaluates the association of each taxon with specific groups of samples (such as habitat types or experimental conditions) based on its consistency and exclusivity within that group (58, 70). Another approach, Threshold Indicator Taxa Analysis (TITAN) (71), is specifically designed for continuous environmental gradients. TITAN identifies taxa exhibiting abrupt changes in abundance at certain thresholds of environmental parameters (e.g., salinity or pollutant concentration), thereby pinpointing not only indicator taxa but also critical points at which community composition shifts occur. This approach has been effectively utilized in freshwater and soil research to detect early-warning signals (72, 73).

Despite significant progress achieved by the methods outlined above, the inherent high-dimensionality, sparsity, zero-inflation, and composition of sequencing data sets limit the effectiveness of traditional methods in accurately identifying specific microbial taxa (26, 74). Moreover, existing practices typically identify keystone, rare, and indicator taxa in isolation, without considering the association and overlaps of these categories, particularly regarding whether rare taxa universally possess keystone properties, and whether keystone taxa may also serve as indicator in specific environments. Due to these limitations, current studies have difficulty capturing the complex interactions of microbial ecological network and the inner contribution of these taxa to the specific ecosystems. These technical challenges and unresolved theoretical gaps in identification continue to impede in-depth ecological inference and hinder the development of effective ecosystem management strategies. To address these challenges, there is an urgent need for advanced analytical tools and systematic frameworks capable of modeling nonlinear relationships, effectively handling data sparsity, and improving the accuracy of identifying microbial taxa (75), thereby paving the way for the adoption of ML methodologies.

## MACHINE LEARNING IN IDENTIFYING MICROBIAL TAXA: CONTRIBUTIONS AND LIMITATIONS

ML has been widely applied in analyzing complex data sets (some classic cases are summarized in Table 1) and uncover microbial coexistent patterns that traditional methods often fail to discern. By leveraging supervised and unsupervised learning techniques, ML facilitates more accurate and scalable approaches to microbial taxonomy, biomarker discovery, and functional inference (33, 75). These applications have demonstrated the robustness of ML in extracting valuable information from sequencing data and outperforming traditional methods in handling complex, high-dimensional data (76–79). As sequencing data accumulates and methodologies advance, researchers have begun to explore the use of ML methods to further identify taxa that have special effects on community ecological functions from massive microbiome data (80–82).

**TABLE 1:** Examples of common tasks and machine learning methods used in microbiome research^*a*^^,^^*b*^

Task	Predictive goal	Method	Data source	Validation strategy	Reference
Specific taxon identification	Keystone taxa	DKI framework	Hybrid	Wet-lab (synthetic community)	Wang et al. (83)
		Linear discriminant analysis	Simulated	Additional simulations	Berry and Widder (40)
		Random forest	Observed	Computational evaluation (genome-resolved analysis)	Cheng et al. (81)
			Hybrid	Wet-lab (invasion experiments)	Wu et al. (84)
		LIMITS	Hybrid	Additional simulations	Fisher and Mehta (85)
	Rare taxa	Ulrb	Observed	Computational evaluation	Pascoal et al. (86)
	Indicator taxa	Random forest, neural networks	Observed	Computational evaluation (independent test set)	Thompson et al. (87)
		Random forest	Observed	Wet-lab (m-ddPCR validation)	Zheng et al. (88)
		Compare supervised ML models (SVMs, RF, linear regression)	Observed	Computational evaluation (genome-resolved analysis)	Liu et al. (89)
		Deep neural networks	Observed	Computational evaluation (comparison with IndVal)	Tsai et al. (90)
Phenotyping	Disease (inflammatory bowel disease)	Random forest, Lasso, elastic nets	Observed	Computational evaluation (cross-study validation)	Wirbel et al. (91)
	Colony morphology classification	Random forest	Observed	Wet-lab	Huang et al. (78)
Interaction analysis	Classification of interactions	Random forest	Hybrid	Wet-lab and additional simulations	DiMucci et al. (92)
Biomarker discovery	Antimicrobial candidates against *E. coli*	Deep neural networks	Observed	Wet-lab (*in vitro* and *in vivo*)	Stokes et al. (93)
Microbial classification	Disease (e.g., colonic screen-relevant neoplasias)	Compare supervised ML models (e.g., SVMs, RF, XGBoost)	Observed	Computational evaluation	Topcuoglu et al. (94)
Functional annotation	Antibiotic resistance genes	HMD-ARG	Observed	Wet-lab (heterologous expression)	Li et al. (95)
Dynamics prediction	Predict future complex community behavior	LSTM	Observed	Wet-lab	Baranwal et al. (96)
Metagenome assembly	Contaminant removal	Deepurify	Hybrid	Additional simulations	Zou et al. (97)
	Metagenomic binning	SemiBin	Observed	Computational evaluation	Pan et al. (98)

^*a*^ Data source indicates whether studies used observed, simulated, or hybrid data. Observed: real-world sequencing data sets (e.g., 16S rRNA amplicon or metagenomics) obtained from natural environments or clinical samples. Simulated: synthetic data generated mathematically (e.g., using generalized Lotka-Volterra models). Hybrid: studies that utilize both real-world observed data sets and simulated data sets (either combined for augmentation or used in parallel for comprehensive benchmarking).

^*b*^ Validation strategy including wet-lab, additional simulation, or computational evaluation. Wet-lab: findings verified through subsequent laboratory experiments (e.g., culturing, qPCR, or synthetic community construction). Additional simulations: validation performed by new data sets under varying parameters or theoretical conditions (e.g., interaction strengths or noise levels) to rigorously test the model’s robustness and theoretical generalizability. Computational evaluation: validation performed solely using statistical metrics (e.g., AUC, precision-recall) or data splitting (e.g., cross-validation) without new experimental data.

ML could serve as a powerful standalone tool that directly leverages sequencing-derived community profiles to identify and delineate ecologically critical taxa. A representative application used random forest (RF) models to investigate the stability of soil microbiomes under disturbance, identifying key metabolic functions and associated microorganisms that contribute to maintaining community stability (82). Among these, specific metabolic pathways, particularly nitrogen metabolism, were identified as “key functions” for maintaining stability, predominantly carried out by certain taxa such as *Nitrospira*. Without relying on network or simulated dynamics, this supervised ML model quantified the contribution of each feature to observed stability outcomes (via feature and permutation importance), thereby highlighting candidate keystone taxa together with the metabolic functions. Similarly, in agricultural microbiomes, ML models help identify key taxa that influence soil microbial community assembly and interactions (99). In addition to feature importance, researchers perform prediction degradation analysis (PDA), which involves removing taxa from community data or randomizing their values to observe the decline in model performance or changes in predicted output to evaluate the impact of taxa on community stability (100–102). This process is actually implemented by permutation importance in the RF model, which is similar to ecologists removing a taxa in an experiment to see if the ecosystem changes occur (103, 104). In summary, supervised ML models assess taxa by their contribution to predicting community-level outcomes, benefiting from using real functional or stability data as a benchmark, rather than relying solely on network structure (22). However, it requires appropriate measurable outcomes or proxies for “community impact” to train models (24).

Accordingly, researchers integrated machine learning with classical identification approaches and reduced spurious associations via reciprocal validation, thereby improving robustness. Berry and Widder (40) combined microbial co-occurrence network topology features with linear discriminant analysis (LDA) based on ML to classify specific taxa according to their network connectivity characteristics. Cheng et al. (81) identified highly connected “hub” taxa in plant rhizosphere microbial communities through co-occurrence network analysis and then verified the accuracy by training multiple ML models. Notably, the RF model highlighted the same taxa (e.g., *Nitrospiraceae*, *Xanthomonadaceae*, *Mycobacteriaceae*) marked as hubs by network analysis, confirming that these taxa were strong predictors of soil potential function, outperforming abiotic factors in explaining soil functional variation. Although the combination of network analysis and ML goes beyond simple hub identification, it also faces challenges in network inference accuracy and model interpretability (44, 105). Another integrative strategy combines classical ecological mechanistic models with ML through computational simulations (84, 85), and the embedding of ecological prior knowledge effectively improves the interpretability of the model. A landmark study by Fisher and Mehta (85) introduced the LIMITS (Learning Interactions from Microbial Time Series), a sparse regression method with bootstrap aggregation. Unlike traditional methods, they inferred Lotka-Volterra species interaction models from human gut microbiome time-series data and successfully identified the keystone taxa that significantly impacted community structure (85). Their key roles in the model dynamics were examined by removal of these taxa from network; however, it requires relatively rich longitudinal data to fit the model. Wu et al. (84) built a microbial community invasion scenario data set based on generalized Lotka-Volterra (gLV) model, offering a novel analytical strategy for data-scarce scenarios by synthesizing labeled invasion scenarios from mechanistic knowledge. Then, they trained an RF classifier to learn a generalizable mapping and identify the most effective “key resistance taxa” by introducing invasive taxa, with predictions transferring to fecal-derived synthetic communities in wet-lab tests (84).

ML also provides novel solutions to address limitations associated with traditional methods for identifying rare microbial taxa. Pascoal et al. (86) introduced “ulrb,” an unsupervised learning-based approach that applied k-medoids to the within-sample abundance vector and partitions taxa into rare, intermediate, and abundant categories without an artificial threshold. As the clustering operates along a single axis of abundance, standard data transformations preserve the relative distances used for classification of compositional and non-compositional inputs. Across distinct sequencing strategies, taxonomic units, and wide ranges of sample sizes, numbers of taxa, and sequencing depths, ulrb yielded consistent rarity assignments and showed better cross-method consistency than fixed-threshold rules. However, ulrb does not explicitly model zero inflation or remove compositional artifacts, as it classifies taxa from within-sample abundance distributions rather than via generative count models. Beyond the abundance classification, ML methods have also been utilized to uncover the functional or interactions of rare taxa (16, 40). Pust and Tümmler (106) revealed that rare taxa were the most critical variables differentiating healthy children versus children with cystic fibrosis (CF) by constructing a classification model. Additionally, anomaly detection algorithms have been employed to identify statistically rare yet highly active or strongly interactive taxa from microbial data sets (107). Ishiya and Aburatani (107) demonstrated that even extremely low-abundance taxa exhibit abundance fluctuations that could signify important ecological shifts or environmental influences. In summary, ML has enhanced the objectivity of rare taxa identification through adaptive abundance thresholds and clustering techniques and also provided valuable approaches to elucidate the ecological roles of these taxa within communities. Nonetheless, due to the inherently low counts, compositional constraints, and high noise associated with rare taxa, accurate identification and importance evaluation remain challenging, and current unsupervised approaches represent an initial step toward comprehensive understanding (61).

Leveraging supervised learning and permutation-based attribution, ML prioritized cross-validated predictive contribution rather than correlation alone, providing a new pathway for identifying indicator. Thompson et al. (87) used indicator species analysis (ISA) to identify 285 bacterial operational taxonomic unit (OTUs) from a total of 1,709 OTUs that significantly correlated with high dissolved organic carbon (DOC) concentrations. By comparing these indicator with those identified as important by RF and neural network models, the researchers found that a subset of 86 overlapping OTUs provided the most robust prediction of DOC concentrations. These overlaps showed that the indicator identified by supervised ML was both statistically significant and predictively informative across heterogeneous communities. Furthermore, supervised ML models have been widely utilized to directly pick biomarkers from abundance data that best differentiate environmental or phenotypic categories (108–111). Zheng et al. (88) employed RF to classify gut microbiome, identifying microbial biomarkers that effectively distinguished inflammatory bowel disease (IBD; including ulcerative colitis [UC] and Crohn’s disease [CD]) patients from healthy controls. Cheng et al. applied an RF model to accurately distinguish the differences in rhizosphere microbiome communities between two rice varieties, identifying family-level indicator taxa as key indicator groups associated with differential cadmium accumulation between cultivars (81). In another study involving anaerobic fermentation reactors, RF regression with Synthetic Minority Oversampling Technique (SMOTE) balancing and fivefold cross-validation accurately predicted product yields in a medium-chain fatty acid-producing system, successfully identified four genera as indicator microbial groups associated with high-yield conditions (89). Collectively, these cases demonstrate that ML models not only predict environmental or functional states but also leverage feature selection processes to uncover the local microbial indicator taxa.

Although ML has been preliminarily applied to identify specific microbial taxa and has yielded valuable insights, it still faces several challenges (44, 112). A fundamental constraint is the heavy reliance on manual feature engineering, which requires substantial domain-specific expertise (74, 79) and frequently introduces biases (30). Handling the high variability of microbial communities across different habitats poses additional challenges for manual feature engineering, as distinct data sets often necessitate entirely different feature sets, thereby restricting the scalability and generalizability of ML models across diverse ecosystems (25, 74, 76). Despite demonstrating superior performance over traditional methods in dealing with high-dimensional data, ML models remain vulnerable to issues such as data sparsity and zero-inflation, which can lead to overfitting or reduced generalization capabilities (113). Furthermore, ML models encounter significant difficulties in integrating multimodal data sets, resulting in insights that are often incomplete or fragmented, thus limiting their capacity to fully elucidate microbial community dynamics (114). Another critical challenge associated with applying ML in microbial research is model interpretability (94). Despite achieving high accuracy in classification and prediction tasks, these models typically function as “black boxes,” obscuring the biological relevance of their outputs (77, 105). This opacity complicates the translation of computational findings into actionable insights, particularly when aiming to understand the mechanisms that drive microbial community structure and functionality (102, 115, 116). These challenges emphasize the pressing need for more effective computational approaches capable of modeling the complex, nonlinear, and hierarchical relationships inherent in microbial data.

## THE RISE OF DEEP LEARNING: A NEW FRONTIER IN IDENTIFYING MICROBIAL TAXA

DL has emerged as a transformative approach to address the limitations of classical ML methods, such as reliance on manual feature engineering and insufficient scalability when handling complex data sets (117). One of the primary strengths of DL lies in its ability to autonomously extract features directly from data without requiring researchers to predefine them, enabling the modeling of intricate nonlinear and hierarchical relationships (118). DL models such as convolutional neural networks (CNNs) have been successfully applied to the classification of microbial taxa based on DNA sequences, achieving impressive accuracy that surpasses traditional methods (119–121). A notable example is the Data-driven Keystone Species Identification (DKI) framework (83), which trains a composition Neural ODE (cNODE2) on steady-state microbiome samples to learn the mapping from species assemblages to compositions and then conducts virtual species removals to compute community-specific structural and functional keystoneness from predicted post-removal changes. DKI recovered true keystoneness on gLV-simulated communities across network connectivities with high accuracy (Spearman ρ ≈ 0.96–0.98) and, in an eight-species synthetic consortium, outperformed topological baselines in wet-lab tests (accuracy 0.85 vs 0.40 for degree and 0.25 for betweenness). Additionally, graph-based DL has gained attention by integrating network analysis with neural networks to identify keystone taxa within microbial communities (122). These applications underscore the critical role of DL in facilitating the rapid and accurate identification of keystone taxa.

The application of DL on multi-omics data analysis has further advanced microbiome research, including the prediction of metabolic pathways, identifying biomarkers, and assessing microbial responses to environmental stressors (30, 32, 123). For example, deep neural network models were trained on tens of millions of promoter-expression pairs to learn an environment-aware sequence-to-expression mapping, enabling accurate cross-condition expression prediction and revealing condition-specific regulatory signals (124). Tsai et al. (90) introduced deep optimal feature learning to identify indicator across ecological habitats, learning low-dimensional representations from 16S rRNA amplicon profiles to prioritize taxa and reveal robust environment-taxon associations. The method was reported to maintain performance under stratified cross-validation, and the indicator sets were more reproducible than traditional feature selection. Attention mechanisms (125), a key architectural innovation in DL, help the model identify which input features matter most in complex data sets by assigning a weight to each candidate taxon, gene, or interaction, thereby facilitating deeper interpretations of microbial interactions and functional roles (126). Melnyk et al. (127) combined microbial network analysis with Transformer-based DL models and applied the interpretability technique of layer-wise relevance propagation (LRP) to elucidate model predictions. By examining the attention weights and relevance scores of the models, researchers were able to highlight which taxa within the community were the most influential in driving transitions from healthy to diseased states.

Identifying rare taxa presents a unique challenge, as signals from these taxa are extremely weak (reflected in low read counts or abundance values) and often obscured by noise and more abundant taxa (61, 128). Traditional methods and classical ML models frequently struggle with class imbalance and low signal-to-noise ratios (details of the cases and discussions are given above in Identifying Specific Microbial Taxa from Meta-Data: Current Status and Challenges and in Machine Learning in Identifying Microbial Taxa: Contributions and Limitations), resulting in ecologically significant rare taxa being overlooked (129, 130). DL approaches offer new strategies to address this issue, one of which is to use DL to learn from massive unlabeled data and denoise complex inputs (121). Autoencoders, for example, have been applied to microbiome data sets to compress high-dimensional community features into lower-dimensional representations (131), effectively filtering out noise while preserving key variation, thereby enhancing the detectability of signals from rare taxa (132). Another powerful approach involves the use of deep generative models for data augmentation to mitigate the paucity of rare taxa examples. Generative adversarial networks (GANs) and related generative models have been utilized to increase model robustness by alleviating class imbalance and expanding the training data sets for rare taxa (133, 134). Early successes have demonstrated that reliable detection of members of the “rare biosphere” is achievable without the need for arbitrary abundance thresholds (86).

 and 3), resulting in ecologically significant rare taxa being overlooked (129, 130). DL approaches offer new strategies to address this issue, one of which is to use DL to learn from massive unlabeled data and denoise complex inputs (121). Autoencoders, for example, have been applied to microbiome data sets to compress high-dimensional community features into lower-dimensional representations (131), effectively filtering out noise while preserving key variation, thereby enhancing the detectability of signals from rare taxa (132). Another powerful approach involves the use of deep generative models for data augmentation to mitigate the paucity of rare taxa examples. Generative adversarial networks (GANs) and related generative models have been utilized to increase model robustness by alleviating class imbalance and expanding the training data sets for rare taxa (133, 134). Early successes have demonstrated that reliable detection of members of the “rare biosphere” is achievable without the need for arbitrary abundance thresholds (86).

Overall, DL methods have overcome many limitations of earlier approaches, enabling more accurate identification of specific microbial taxa. However, despite these significant advantages, there remain several key challenges to the broader application of DL in identifying specific microbial taxa. Issues such as overfitting, data bias, and class imbalance hinder the robustness and generalizability of DL models (118, 135–137). Additionally, the “black boxes” nature of DL models raises concerns about interpretability and reliability. Although tools such as Shapley additive explanations (SHAP) and local interpretable model-agnostic explanations (LIME) have been developed to elucidate DL predictions, ensuring their biological relevance remains an urgent task (138, 139). Furthermore, the computational cost and hardware requirements of DL models are typically high, raising concerns about scalability and deployment in real-world applications (140). Nevertheless, the rise of DL represents a paradigm shift in microbial taxa identification, providing unparalleled tools for analyzing complex data sets and modeling dynamic interactions within microbial ecosystems. As advances continue in data standardization, model interpretability, and computational infrastructure, DL holds the potential to drive transformative innovations in microbial research, spanning health, environmental sustainability, and biotechnology.

## FUTURE DIRECTIONS OF MICROBIAL TAXA IDENTIFICATION BY DEEP LEARNING

### Conceptual foundations

**Fig 1:**
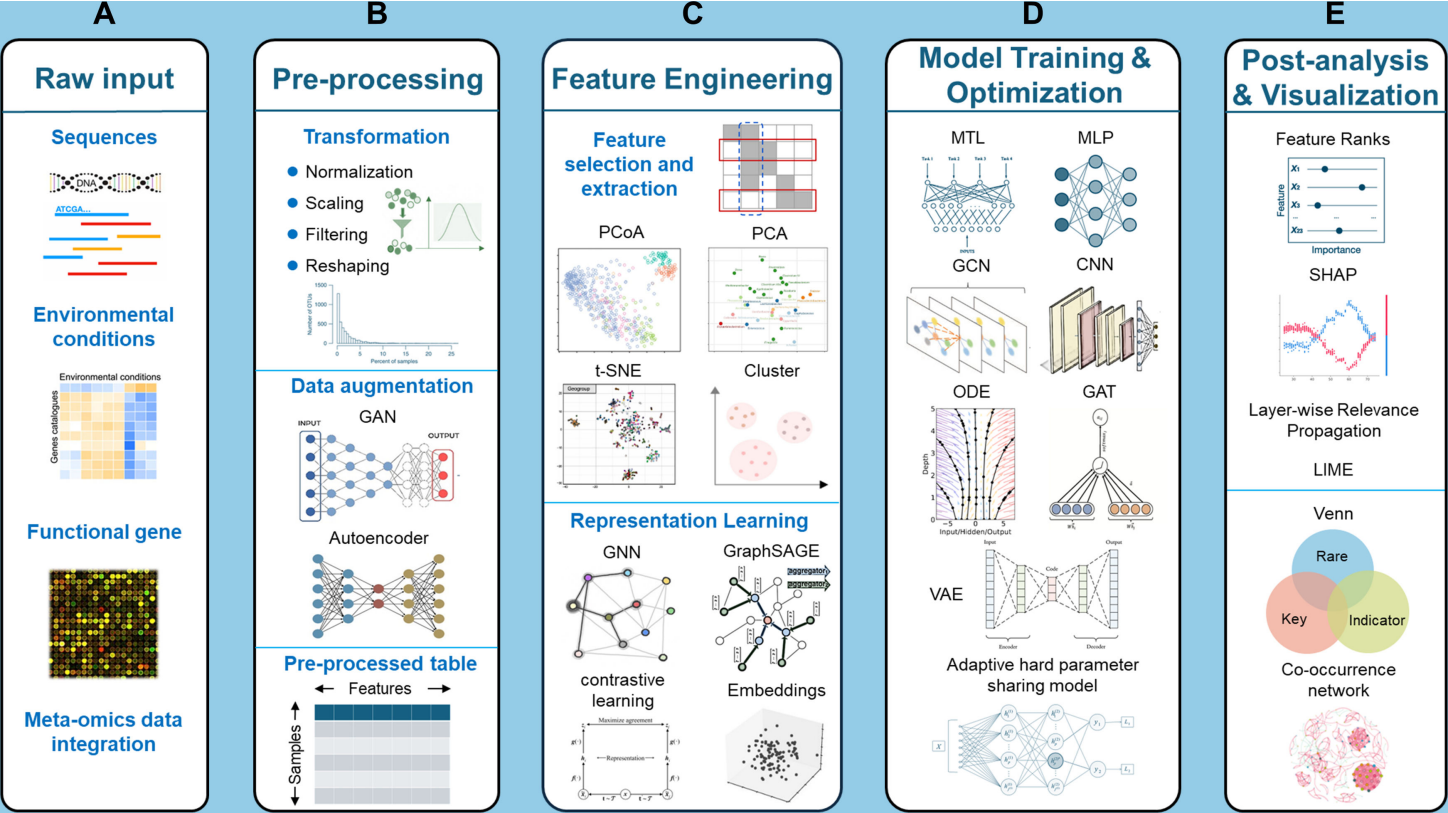
Overview of a unified deep learning framework for microbial taxa identification. (**A**) Raw input, define the range and resolution of the data. (**B**) Pre-processing, eliminate technical artifacts and improve comparability, preparing for data analysis. (**C**) Feature Engineering, convert data tables into structural feature information. (**D**) Model Training & Optimization, learn predictive mappings. (**E**) Post-analysis & Visualization, explain and validate. Abbreviations**:** MLP, multilayer perceptron; CNN, convolutional neural network; GCN, graph convolutional network; GAT, graph attention network; ODE, neural ordinary differential equation; VAE, variational autoencoder; SHAP, Shapley additive explanations; LRP, layer-wise relevance propagation; GAN, generative adversarial network; PCoA, principal coordinates analysis; PCA, principal component analysis; t-SNE, t-distributed stochastic neighbor embedding; CLR/ALR, centered/additive log-ratio; ZINB, zero-inflated negative binomial.

The application potential of DL in identifying specific microbial taxa has been initially demonstrated, showcasing its robust capabilities in analyzing complex microbial ecosystems (83, 86, 90). Despite these advances, these approaches are typically task-specific or tailored to individual ecological contexts, limiting their broader applicability. In addition, current DL applications are mainly focused on amplicon and shotgun metagenomic data sets, and comprehensive utilization of multi-omics data sets for holistic microbial community studies remains to be further explored (33, 141). Against this backdrop, we propose a future direction for DL in identifying specific microbial taxa: a unified multitask framework based on multi-modal data integration that simultaneously identifies keystone, rare, and indicator taxa (Fig. 1). This unified framework aims to efficiently identify these three categories of taxa through standardized data processing and analytical pipelines, uncovering their interrelationships and ecological significance within microbial communities. By combining the ecological functions of keystone, the dynamic characteristics of rare taxa, and the environmental responses of indicator, this direction offers promising opportunities to explore the intricate complexities of microbial community structure and function.

### Framework design

This section presents the modular blueprint of a unified framework for identifying ecologically important taxa in microbiomes. It focuses on the core design challenges and explains, in a layered manner, the construction logic and technical selection of each module. From data preprocessing and augmentation (“Data preprocessing and augmentation”), to compositional and hierarchy-aware representations (“Feature engineering and representation learning”), to multi-paradigm model architectures for prediction (“Multi-paradigm model architecture”), and finally to interpretability, validation, and communication with multi-omics integration (“Interpretability validation and optimization”), the aim is to provide a reusable analysis workflow. Additionally, to help policymakers and practitioners intuitively understand how deep-learning “black-box” models operate, we provide a plain-language explainer (see Table 2).

**TABLE 2:** A plain-language guide to machine learning workflow in microbial ecology

Key aspect	Plain-language explanation
What data are used as input	Data on one or more categories of taxa and their abundances, optional genes and pathways, metabolites, and environmental or clinical variables
What does model training refer to	The model sees many labeled examples and adjusts internal weights so that similar patterns lead to similar outcomes
What does a prediction represent	For a new sample, the model computes a score or class that reflects patterns it has seen before
How do we know what matters	Metrics such as contribution scores (e.g., SHAP) and simple attention weights are aggregated to show which taxa, genes, or pathways were most influential for that sample
How do we validate results	By comparing potential (DNA), activity (RNA or proteins), and outputs (metabolites), and testing with independent test data, reporting uncertainty when confidence is low
What are the limitations of these models	These models reveal associations within the observed data. But they do not prove causation and they depend on data quality, sampling depth, and preprocessing choices

#### Data preprocessing and augmentation

Microbiome data preprocessing constitutes the foundational phase of framework construction (Fig. 1A and B). Because amplicon and metagenomic abundance tables are inherently sparse, subject to batch effects, and commonly zero-inflated, preprocessing should implement multi-stage correction and augmentation, including robust zero handling and a log-ratio transformation (centered log-ratio or additive log-ratio for targeted contrasts). For multi-omics analyses, preprocessing should also align sample identifiers and time points across data layers and construct layer-specific feature matrices with appropriate normalization, such as log-ratio features for compositional abundance layers and standard scaling procedures for expression and metabolite intensities. To address zero-inflation artifacts, the zero-inflated negative binomial (ZINB) model employs a mixture distribution framework to distinguish technical zeros (induced by insufficient sequencing depth) from true ecological zeros (reflecting actual taxa absence) (142, 143). The dual-component structure of this model has demonstrated superior performance in microbiome data analysis (144). For cross-data set integration scenarios, the ComBat algorithm based on empirical Bayesian estimation systematically eliminates technical biases introduced by heterogeneous sequencing platforms or experimental conditions through parameterization of batch-specific offsets (145), with its standardization process proving particularly advantageous for multicenter microbiome studies. To further mitigate underrepresentation of rare taxa, GANs synthesize ecologically plausible low-abundance samples via adversarial training between generator and discriminator modules (133, 146). In the human gut microbiome, GAN-based augmentation has produced realistic community profiles and improved downstream classification and cross-cohort generalization (147, 148). Taken together, this methodology has exhibited promising potential for data augmentation in gut microbiota rare taxa identification. The synergistic application of these preprocessing and augmentation steps produces harmonized, analysis-ready inputs for the representation, modeling, and interpretability stages that follow.

#### Feature engineering and representation learning

Extracting ecologically meaningful low-dimensional representations from high-dimensional OTU tables represents a pivotal challenge in framework design (Fig. 1C). Conventional approaches such as ANCOM-II (Analysis of Compositions of Microbiomes) employ log-ratio transformations coupled with multiple hypothesis testing to identify differentially abundant OTUs across experimental groups (149). However, their linear assumptions inadequately capture complex taxa interaction patterns (150, 151). Consequently, graph neural network-based embedding methods have emerged as a transformative solution. By constructing OTU co-occurrence networks through SparCC (Sparse Correlations for Compositional data) correlation analysis (45), the GraphSAGE (Graph Sample and Aggregate) model learns topological features of nodes within microbial interaction networks (152), where neighborhood aggregation mechanisms effectively encode synergistic or competitive relationships among taxa. Furthermore, contrastive learning techniques maximize representation consistency between OTU tables and environmental covariates (153), embedding niche theory into feature spaces to automatically associate microbial composition with host habitat characteristics. When multi-omics data are available, representation learning also benefits from layer-specific features and simple cross-layer links. Compositional abundance layers are expressed with log-ratio features, gene and pathway profiles are aggregated from metagenomes, and standardized expression or metabolite intensities are derived from metatranscriptomes, metaproteomes, and metabolomes. Two practical cross-omics features are the activity-to-abundance ratio, for example, RNA over DNA for a taxon or pathway which highlights low-abundance high-activity patterns relevant to rare taxa, and gradient-concordance vectors that summarize how taxa, pathway activity, and metabolites co-vary with an environmental or clinical gradient for indicator taxa. Joint or contrastive embeddings can then align the taxon space with pathway and metabolite spaces so that the learned manifold reflects ecological niches and functional constraints. The integration of these methodologies constructs discriminative and interpretable feature spaces that enable high-quality inputs for multitask modeling.

#### Multi-paradigm model architecture

The framework adopts a multi-paradigm fusion strategy at the architectural level to address ecosystem complexity (Fig. 1D). Multitask learning (MTL) extracts cross-task invariant features through shared encoders while employing task-specific modules for simultaneous prediction of keystone, rare, and indicator taxa (154). This hard parameter-sharing mechanism reduces overfitting risks while enhancing model discriminative capacity for ecological functional groups (155). With multi-omics input, the architecture can use early fusion by concatenating standardized features or late fusion with per-omics encoders that feed a shared trunk. These distinct modules for keystone, rare, and indicator taxa allow attention or gating to reveal which data layer supports each decision. For dynamic community assembly processes, neural ordinary differential equations (Neural ODEs) integrate Lotka-Volterra dynamics into differentiable solvers (156), enabling temporal inference of taxa interaction networks from static snapshot data (157). In practice, trajectories can be constrained by short time series or pseudo-time, and the model can produce time-dependent interaction estimates together with dynamic importance profiles for taxa and pathways. In spatial-scale analyses, graph attention networks (GATs) utilize adaptive weight allocation mechanisms to capture heterogeneous interaction patterns within OTU co-occurrence networks (158). When multi-omics information is available, graphs can be extended to multi-relational forms in which edges encode co-occurrence, pathway linkage, or metabolite similarity, and attention weights highlight candidate interactions within and across layers. The coordinated design of these models equips the framework to resolve both steady-state ecosystem properties and simulate temporal community dynamics.

#### Interpretability validation and optimization

The framework systematically identifies taxa that span multiple categories (159). These overlapping taxa, which may simultaneously act as keystone, rare, and indicator taxa, represent a compelling area of study. To further elucidate the ecological significance of these taxa, the framework establishes a multimodal validation system that integrates explainable artificial intelligence (XAI) techniques (e.g., LRP [160], SHAP, or LIME). By integrating visualization tools such as Venn diagrams, co-occurrence networks, and heatmaps (Fig. 1E), this system visually translates computational patterns into ecological insights; specifically, the co-occurrence networks here are used to depict model-inferred non-linear dependencies (e.g., attention weights or interaction coefficients) rather than simple statistical correlations (83, 126, 127). When working with multi-omics data, attribution is first computed within each omics layer and then aggregated along the links of taxa, genes, pathways, and metabolites to form a cross-layer evidence profile for each taxa. SHAP quantifies contributions at the OTU level through cooperative game theory (116, 138), and the same procedure is applied to genes, pathways, and metabolites. Model importance can be linked to functional potential, realized activity, and pathway output in a multi-layer evidence graph. Taxon is prioritized when evidence is consistent across layers, such as keystone showing high model importance accompanied by pathway activity or metabolite changes that may affect stability. Rare taxa show low DNA abundance together with elevated RNA or protein activity or a distinct metabolite footprint. Indicator exhibits taxa, pathway activity, and metabolites that co-vary in consistent directions across cohorts along environmental or clinical gradients.

Synthetic community construction is used to validate model-predicted specific taxa and to assess community stability, establishing closed-loop feedback between computational predictions and laboratory observations (161). During model update and optimization, the adoption of dynamic curriculum learning strategies that progressively increase data complexity (e.g., incremental learning from dominant to rare taxa) (162) can improve the ability of the model to fit long-tailed distributions and training efficiency (163) without changing the model architecture. In addition, the temporal perspective can be incorporated to study microbial community assembly across different habitats and gradients (164), enabling systematic dissection of how the roles and interactions among keystone, rare, and indicator taxa evolve over time in response to environmental perturbations, disturbance regimes, or successional trajectories (59, 165). The systematic integration of these validation mechanisms contributes to a more comprehensive understanding of microbial ecosystem dynamics, thereby ensuring theoretical compatibility between framework outputs and ecological principles.

### Core challenges and theoretical bottlenecks

The construction of current DL frameworks remains confronted with several cross-cutting challenges arising from fundamental tensions between biological system complexity, intrinsic data characteristics, and computational model capabilities. The primary challenge stems from the conflict between the heterogeneous, low signal-to-noise nature of microbiome data and the generalization capacity of models. Although existing preprocessing techniques (e.g., batch effect correction [166], data augmentation [167]) have partially mitigated data quality issues, the high dimensionality, sparsity, and multimodal characteristics of microbiome data continue to challenge model robustness. For instance, signals from low-abundance taxa are often faint and difficult to distinguish from technical noise. This signal overlap makes it challenging for models to discriminate between spurious statistical correlations and genuine biological associations (168). Furthermore, the lack of standardization across studies (e.g., inconsistencies in sampling protocols and sequencing depths) induces fundamental shifts in the underlying data distribution (feature space shifts) during pretrained model transfer (112, 169), which often causes models trained on one data set to fail when applied to another. Consequently, this necessitates the development of meta-learning architectures capable of projecting diverse data into universal representation spaces, where biological signals from diverse data sets of different studies are aligned and conserved.

A second pervasive challenge lies in balancing ecological interpretability with predictive accuracy across framework hierarchies. While DL models excel at capturing complex nonlinear relationships, their opaque decision-making mechanisms often create explanatory disjunctions between computational logic and ecological theory (170), particularly in causal inference of host-microbiome interactions (171). Current interpretability methods (e.g., feature importance ranking) provide OTU-level contribution analyses but fail to elucidate ecosystem-scale principles such as multi-taxa synergies or temporal succession dynamics (172, 173). A more fundamental tension arises from the inability of current discriminative objective-driven DL paradigms to holistically model emergent properties of microbiome systems (e.g., community stability, functional redundancy), demanding hybrid architecture-inference systems that integrate first principles of ecology.

Finally, the mismatch between computational resource constraints and the inherent complexity and multi-scale nature of biological systems limits the practical boundaries of frameworks. Microbiome studies frequently involve ultrahigh-dimensional OTU features (≥10^4^ dimensions) (174) and multiple temporal scales (ranging from minute-level metabolic dynamics to decadal ecological succession) (175), posing dual challenges to model memory efficiency and long-term dependency modeling. Traditional DL approaches face a dilemma between the “curse of dimensionality” (i.e., too many microbial features with too few samples lead to a decrease in model performance) and “over-smoothing effects” (i.e., where distinct nodes in a graph neural network become too similar to distinguish) when addressing such problems—increasing network capacity enhances feature discrimination but exacerbates overfitting on scarce annotated data (118). Breakthroughs in physics-informed neural networks (PINNs) and sparse training strategies are urgently required to overcome computational bottlenecks (176, 177). Simultaneously, orders-of-magnitude disparities between synthetic biology techniques (for wet-lab validation), microbial cultivation cycles, and computational model iteration speeds hinder efficient coordination of “dry-wet closed loops” (161, 178, 179). To overcome these disparities, employing active learning strategies to iteratively select the most informative experimental validations can minimize the wet-lab workload. Anyway, overcoming these technical challenges is key to fully unlocking the potential of DL in advancing microbial ecology and broader biological research.

## FUTURE PROSPECTS

Future developments should prioritize the integration of multi-modal data sources to capture the complete dynamics of microbial communities across genetic, transcriptional, and metabolic levels, thereby enriching ecological insights (180). The development of DL frameworks must also emphasize enhancing model interpretability by incorporating causal inference capabilities. The inclusion of dynamic feedback loops could provide a more accurate means to simulate temporal variations in microbial communities (157), capturing their responses to long-term evolutionary dynamics, including successional processes and environmental disturbances. Furthermore, to improve the accessibility and applicability of DL frameworks, it is essential to design more efficient and lightweight computational architectures. Simultaneously, advancing community-wide standardization of data formats and ensuring transparency in framework design (e.g., open source, documentation, and clearly defined application boundaries) will be critical for fostering consistency and reproducibility in future research (181, 182).

## CONCLUSION

Deep learning is driving a transformative shift in microbial taxa identification from communities, offering novel perspectives through automated feature extraction and the modeling of complex nonlinear interactions. This review examines the progression of identifying specific microbial taxa, from traditional methodologies to the advancements brought by deep learning, emphasizing the paradigm shift enabled by these innovations. The categories of keystone, rare, and indicator taxa are dynamic and context-dependent, influenced by environmental gradients, community interactions, and functional redundancy. A unified deep learning framework was proposed by integrating the identification of keystone, rare, and indicator taxa into a cohesive computational workflow. Bridging the gap between microbial ecological theory and data-driven computational approaches using multi-task learning to model shared ecological patterns while preserving task-specific nuances. By harmonizing deep learning with ecological theory, it offers a powerful tool to unravel the complexities of microbial communities, with applications spanning environmental monitoring, biotechnology, and human health. Future work will focus on empirical validation across biomes and the development of user-friendly pipelines for broader adoption.
